# Mental Health Prevention in Preschool Children: study protocol for a feasibility and acceptability randomised controlled trial of a culturally adapted version of the I Can Problem Solve (ICPS) Programme in Chile

**DOI:** 10.1186/s13063-019-3245-3

**Published:** 2019-03-04

**Authors:** Jorge Gaete, Marcelo Sánchez, Lorena Nejaz, Mikele Otegui

**Affiliations:** 1 Department of Public Health and Epidemiology, Faculty of Medicine, Universidad de los Andes, Santiago, Chile; 2 San Carlos de Maipo Foundation, Santiago, Chile

**Keywords:** Prevention, Promotion, Mental health, Preschool, Problem behaviour

## Abstract

**Background:**

Difficulties with delaying gratification, coping with frustration, and regulating emotions are significant predictors of aggression and behavioural and interpersonal problems early in life and mental health disorders during childhood, adolescence, and adulthood. Mental health problems generate a high burden of disease in society in general, and there is a significant treatment gap, especially among economically vulnerable populations. Prevention strategies appear to be the more recommendable options, mainly if these interventions can be implemented early in life and at low cost. Few preventive interventions aiming to increase resilience in the face of adversity have been rigorously evaluated among Chilean preschoolers. Substantial international evidence indicates that strengthening basic psychological skills, such as emotion regulation and social problem-solving, can reduce the incidence of mental pathology and improve various academic indicators. The curriculum of the Interpersonal Cognitive Problem-Solving Programme, also known as I Can Problem Solve (ICPS), is focussed on the development of the cognitive process and children’s social problem-solving skills. ICPS is effective at increasing prosocial behaviours and reducing aggressive behaviour among preschoolers. ICPS provides children with the skills to think about how to solve problems using sequenced games, discussion, and group-interaction techniques focussed on listening to, and observing, others, promoting empathy and alternative and consequential thinking. The aims of this study are (1) to develop a culturally appropriate version of the ICPS programme and (2) to evaluate the acceptability and feasibility of the adapted version of ICPS among vulnerable schools in Santiago, Chile, conducting a pilot randomised controlled trial with three arms: (1) the ICPS programme delivered by an internal early teacher, (2) the ICPS programme delivered by an external early teacher, and (3) a control group.

**Methods and design:**

This is a pilot, three-armed randomised controlled trial of the adapted version of ICPS with an enrolment target of 80 preschoolers attending four schools per arm. Children in both intervention groups will receive the ICPS programme: 59 sessions of 20 min each delivered three times a week by trained internal or external early teachers over 5–6 months. Internal teachers are part of the school staff, and external teachers are facilitators hired by the research team to go to schools and deliver the intervention during a normal school day, working together with the early teacher present in the classroom. The intervention consists of games using pictures, puppets, and simple role-playing techniques to facilitate the learning process. Cognitive regulation, emotion recognition, social-problem-solving skills, and psychological functioning will be measured at baseline and after the intervention.

**Discussion:**

No previous studies in Spanish-speaking Latin American countries have been conducted to explore the acceptability and feasibility of ICPS to provide information to evaluate the effectiveness of this intervention on a larger scale.

**Trial registration:**

ClinicalTrials.gov, ID: NCT03383172. Registered on 26 December 2017.

**Electronic supplementary material:**

The online version of this article (10.1186/s13063-019-3245-3) contains supplementary material, which is available to authorized users.

## Background

Mental health disorders are among the leading causes of the Global Burden of Disease [1, 2], and their relative importance is predicted to rise globally. The treatment gap for mental health disorders is large [3], especially in populations that are more economically vulnerable [4]. The ideal strategy to tackle this gap should be that of preventing the onset of these conditions. Most adult mental disorders start in childhood or adolescence and delaying or preventing the onset can have a substantial impact.

In Chile, mental health among children and families is an urgent public health problem. Several epidemiological studies have shown that a significant percentage of the adult population has psychiatric disorders. For example, one recent study reported that 31.5% of the population aged 15 years and older has some type of psychiatric pathology in their life and that 22.2% suffered from a psychiatric disorder during the last year [5]. The few epidemiological studies on the Chilean child and adolescent populations have shown that the prevalence of psychiatric disorders among children of between 4 and 11 years old was 27.8%, a higher percentage than in adolescents of between 12 and 18 years old, which was 16.5% [6]. The most frequent disorders in the population of between 4 and 11 years old were disruptive disorders (20.6%), followed by anxiety disorders (9.2%) [6].

Various studies have shown that children from socio-economically vulnerable families have a higher risk of behavioural difficulties given their exposure to a greater number of risk factors [7]. Among these behavioural problems are disruptive disorders such as behavioural disorder, defiant oppositional disorder, and attention deficit disorder with/without hyperactivity [8]. Many of these children also have a variety of deficits in social-emotional skills [9], and a combination of these disorders puts them at greater risk of having problems with school activities, their peers, and their academic performance [10]. Several studies have shown that poor development of basic psychological skills can increase the incidence of psychiatric disorders and reduce various academic indicators. For example, low emotional regulation [11] and few problem-solving skills [12] has been associated with a higher incidence of depression.

Scientific evidence has recently indicated that the stimulation of cognitive and non-cognitive skills in the first years of life promotes general development and has a beneficial long-term impact on health [13, 14] and on different economic indicators [15–17]. However, much of this evidence comes from studies in the United States, such as the High/Scope Perry Preschool Study [18], the Abecedarian Project [19, 20], Head Start [21–23], and Early Head Start [19–24]. Many of these interventions were costly and difficult to implement.

On the one hand, cognitive skills refer mainly to executive functions, such as attention, working memory, inhibitory control, and cognitive flexibility [25], that are essential for the adequate development of language, literacy, and mathematical problem-solving. Many of these skills are measured through specific measures or standardised tests [26]. On the other hand, non-cognitive skills [27] refer to social-emotional learning (SEL) skills such as solving social problems, the ability to empathise with the needs of others, persistence and the ability to delay gratification, self-regulation, and motivation to learn, among others. There is clear evidence of a mutual relationship between cognitive and social-emotional skills, that is, the development of one favours the development of the other and vice versa [28].

Social and emotional learning skills, as mentioned above, can be categorised into three main groups: cognitive regulation, emotional processes, and social and interpersonal skills. There are alternative ways to categorise them, such as the model presented by the Collaborative for Academic, Social, and Emotional Learning (CASEL) group [29], but these allude to the same functions presented here. In addition, other functions have recently been considered as part of these social and emotional skills, such as character [30], mentality [31], and tenacity [32, 33].

Cognitive regulation is one of the basic skills required to direct behaviour towards the attainment of a specific goal. It is closely related to the concept of executive function and is used for prioritising and sequencing behaviours (for example, putting on socks before shoes), inhibiting one behaviour in favour of a behaviour more appropriate to the context (for example, asking for permission before running to the bathroom), considering relevant information (for example, remembering an instruction from the teacher before starting a task), resisting distractions, changing tasks, using information to make decisions, and creating abstract rules to handle new situations [34]. There is evidence that, in this category, the main skills that have shown positive results in the development of children and young people are attentional control, inhibitory control, working memory, planning, and cognitive flexibility.

Emotional processes are a group of skills that help children to recognise, express, and regulate their emotions [35]. These skills also help to recognise other peoples’ perspectives and emotions. Emotional skills allow children to recognise their resulting emotions triggered in different situations and how to use them in a prosocial manner. These skills also help to build good social relationships with their peers. The skills included in this category are [35] knowledge, awareness and expression of emotions, regulation of emotional behaviour, empathy, and perspective.

Social and interpersonal skills help children to adequately interpret the behaviours of others, manage social situations effectively, and interact with peers and adults. These skills are built on the aforementioned emotional processes. Children must first be able to recognise, express, and regulate their emotions before building effective social relationships. These skills help children and young people to collaborate effectively on their schoolwork (and later in the workplace) and to solve social problems. The main skills in this category are [29, 34] understanding social codes, solving social problems, and prosocial skills.

There is sufficient evidence that shows the benefits of interventions aiming to promote social and emotional skills. A recent meta-analysis reviewed 82 universal school-based interventions involving 97,406 students from kindergarten to high school with a follow-up period of 6 months to 18 years [33]. SEL interventions had a positive effect on several outcomes, with a significant effect size of between 0.13 (attitude improvement) to 0.33 (better academic performance). This is a large effect size for universal interventions.

There are interventions that have proven effective in reducing mental disorders and promoting social-emotional competence [36] such as Incredible Years [10], Promoting Alternative Thinking Strategies (PATHS) [37], and I Can Problem Solve [38]. Each of these interventions has its own advantages and disadvantages; however, none has an available adaptation that has shown efficacy in Spanish-speaking populations of Latin America. Before adopting the widespread use of any programme of extra-cultural origin, it is necessary to make the required cultural adjustments, consider the costs involved, and determine its effectiveness in the context where it is to be implemented [39]. No interventions in Chile using a randomised controlled trial design have proven effective at reducing behavioural problems and promoting mental health among preschool children coming from socio-economically vulnerable families.

I Can Problem Solve (ICPS) was recently selected as one of the top 25 programmes to implement in schools and promote social-emotional skills in a careful review conducted by Jones et al. (2017) [34]. Similarly, several agencies in the United States and Canada, working to identify interventions that show the best levels of evidence for different outcomes, have recognised ICPS as a promising intervention [29, 40–42]. After carefully assessing the technical and economic resources necessary to initiate a process of adaptation and subsequently studying the effectiveness of all of the aforementioned studies, the ICPS programme was selected. This programme is also especially promising since it has demonstrated effectiveness in socio-economically vulnerable populations.

### I Can Problem Solve

The curriculum of the Interpersonal Cognitive Problem-Solving Programme, also known as I Can Problem Solve (ICPS), is focussed on the development of the cognitive process and children’s social problem-solving skills. That is, it is a programme that explicitly promotes cognitive regulation (skills for listening and paying attention, sequencing, and planning tasks), solving social problems (proposing alternative solutions, causal thinking, means-to-an-end thinking, and sequential planning), and the promotion and learning of emotional processes (particularly emotional expression/knowledge, perspective, and empathy). A recent analysis of the included activities [34] that assessed the percentage of activities dedicated to each of these social-emotional skills found that 65% of the activities explicitly aim to develop cognitive regulation, 65% aim to develop emotional processes, and 55% aim to develop interpersonal skills. Programme activities may include tasks that are in more than one domain or category of analysis, so these percentages do not add up to 100%. In terms of the type of activities included in this programme, a high percentage (63%) use discussion groups as a strategy, followed by visual games (23%), role-playing games (23%), interactive games (19%), and vocabulary teaching (15%), mainly in relation to emotions, planning, and sequencing [34].

Two randomised controlled trials tested the effectiveness of ICPS. In the first study [43], a group of preschoolers was studied over a period of 2 years, during nursery school and kindergarten. A total of 113 students were part of the intervention group, and 106 were controls. Overall, 69 students from the intervention group participated for 2 years, while 69 were evaluated in the control group. Four groups were compared: trained nursery-trained kindergartners (TT); trained nursery-control kindergartners (TC), control nursery-trained kindergartners (CT), and control nursery-control kindergartners (CC). A total of 21 teachers were trained. The evaluation used the following questionnaires: (1) Preschool Interpersonal Problem-Solving (PIPS) which directly evaluates problem-solving; (2) the What Happens Next Game (WHNG) which directly evaluates social interactions; and (3) the Hahnemann Preschool Behaviour Scale (HPSB) which evaluates problematic behaviours (such as impulsiveness, inhibition, and adaptation) and was answered by the teacher. A marked difference was found in favour of the intervention group in terms of the improvements related to PIPS (F (1, 213) = 106.90, *p* < 0.001), the WHNG (F (1, 213) = 23.80, *p* < 0.001), and the behaviour adjustment measures (all Fs, *p* < 0.001). The results also show that these effects are sustained over time after 1 year. When comparing the results between well-adjusted, impulsive, or inhibited children, improvements in problem-solving skills were evident in all three groups. Finally, when studying the effectiveness in relation to training time, 2 years (TT) vs 1 (TC and CT) or none (CC), those who received 2 years of training had better results than those who were trained for 1 year, and all of the groups that received training had better results than those that did not (CC).

In a second study [44], participating schools were randomly distributed between an intervention group (ICPS) and a control group. A total of 226 students were assigned to three conditions: ICPS for 2 years (kindergarten and first grade, *n* = 96), ICPS for 1 year (kindergarten or first grade, *n* = 106), and a control group (*n* = 24) without ICPS. The majority of the participants were of Hispanic origin, and more than 90% of the children received free lunches (which is a poverty indicator used in the United States). The student evaluations were based on: (1) the Preschool Social Behaviour Scale (PSBS), which evaluates relational and direct aggressive behaviours in addition to prosocial behaviour, has 16 items, and is answered by teachers; and (2) the Hahnemann Behaviour Rating Scale (HBRS), an instrument that evaluates student behaviour in relation to aggressiveness, impulsiveness, passivity, and prosocial behaviour, and allows for the creation of sub-scales of aggression/impulsivity (for example, hits others, pushes others, and/or angers easily), passivity (for example, shyness and/or social withdrawal), and prosocial behaviour (for example, whether or not other children like them). It consists of 11 items and is answered by teachers. Students who received 2 years of ICPS had the best results in terms of increasing prosocial behaviours and reducing aggressive behaviour. For the HBRS, on the sub-scale of prosocial behaviour, a 12% greater effect was shown for the ICPS group (both groups that received ICPS). But when those who received ICPS for 1 year were excluded from the analysis, comparing only those who received 2 years of ICPS with those who received none, this effect increased by 19% in favour of the intervention group. Similarly, for the PSBS, the sub-scales of open and relational aggression showed 3 and 4% improvement in favour of the intervention, respectively. But when those who received ICPS for 1 year were excluded from the analysis, the effect was 6 and 9%, respectively, in favour of the intervention group.

## Aims and hypothesis

The general objective of this study is to evaluate the acceptability and feasibility of the adaptation of the I Can Problem Solve (ICPS) programme for preschoolers in a national context at educational institutions with high socio-economic vulnerability, and to compare the implementation process and fidelity of the programme delivered by school staff early teachers (internal facilitators) and by external early teachers (external facilitators). The specific objectives are:

1. To adapt the ICPS programme through a process of translation, editing, and cultural adjustment
2. To evaluate the acceptability of this programme, and the instruments it uses, by students, parents, teachers, and authorities at Chilean educational institutions
3. To evaluate the feasibility of implementing this programme and the instruments it uses by studying (1) the participant recruitment process to report on the feasibility of a randomised controlled trial by large-scale clusters, (2) the time and effort required to answer the programme’s evaluation scales, and (3) the measurement of the response rate, loss levels during follow-up, and general information lost in the study
4. To compare the acceptability, feasibility and fidelity of the implementation of this programme between internal and external facilitators
5. To identify and compare changes in social-emotional competence as well as the presence of emotional and behavioural problems among preschoolers in the intervention group schools and the control group schools

The following hypotheses are presented in this study: the hypothesis related to the feasibility and acceptance of this intervention programme (H1) and those related to the expected effects of the intervention (H2 and H3):

- H1: The recruitment and adaptation of the interventions, evaluations, and procedures in this study will be feasible and acceptable in the national context at educational institutions with high socio-economic vulnerability, which will allow progress to be made towards the subsequent proposal of a randomised controlled trial using large-scale clusters
- H2: The acceptability, feasibility, and fidelity of the implementation of this programme will be better in the intervention groups led by the external facilitator
- H3: Preschoolers in the intervention group will have a greater degree of social-emotional competence compared to preschoolers in the control group
- H4: Preschoolers in the intervention group will have fewer emotional and behavioural symptoms compared to preschoolers in the control group

## Methods and design

### Context

Municipal and private subsidised educational institutions of the province of Santiago will be invited to participate. Within the province of Santiago, schools from four municipalities (Estación Central, Peñalolén, Quilicura, and Lo Espejo) will be contacted and invited to participate. Refusal reasons will be noted.

### Design

This is a pilot study and, therefore, primarily exploratory. It will provide data on the acceptability and feasibility of an ICPS programme adapted to the Chilean context and culture. This study also has a quantitative component that will be a single-blind, three-armed randomised controlled trial. Two groups of schools will receive the intervention: (1) in one group, the intervention will be delivered by trained external facilitators, that is, early teachers hired by the research project to go to the schools and deliver the intervention to the students and, at the same time, allow the participation of the early schoolteacher in the ICPS activities; and (2) in the other group, the intervention will be delivered by trained internal preschool educators. Both intervention groups will receive the adapted ICPS programme, while the educational institutions in the control group will continue with their normal activities.

This design will enable the comparison of several aspects of the implementation; for example: how frequently the ICPS programme can be delivered each week over the entire academic year, the fidelity of implementation, and the difficulties faced by external and internal facilitators in order to determine which option may be more practical and feasible for a larger randomised controlled trial (see Additional file 1).

**Fig. 1:**
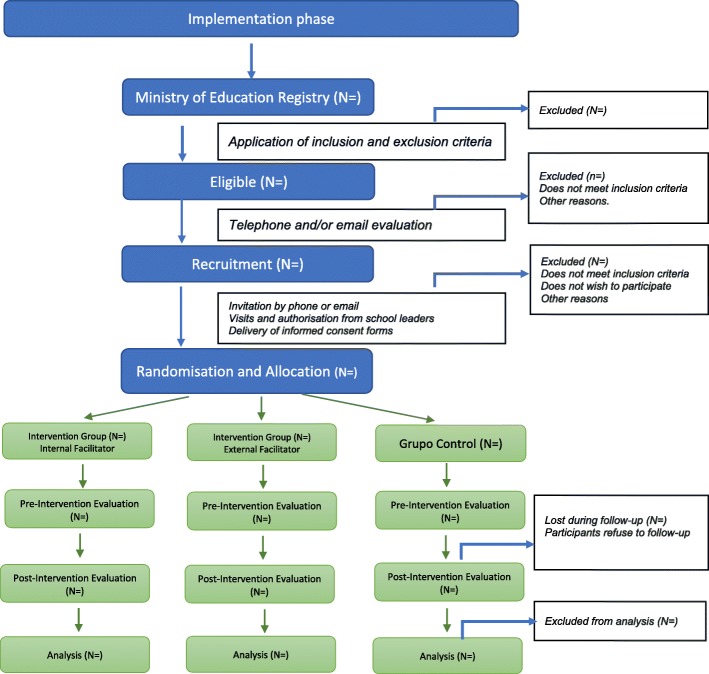
Standard Protocol Items: Recommendations for Interventional Trials (SPIRIT) diagram

Figure 1 shows the Standard Protocol Items; Recommendations for Interventional Trials (SPIRIT) diagram for the trial.

### Participants

The sample will be obtained from the municipal (public) and subsidised (charter) schools of the province of Santiago. Informed consent forms (for each stage of this study, the adaptation/validation of instruments and pilot intervention) will be sent to the parents/guardians explaining the objectives of the research and giving them the option to enter and exit the study whenever they wish. The parents or guardians must provide their written consent in order to participate in the study. For those who agree to participate in the pilot intervention and are selected for the intervention groups, their child will receive the adapted ICPS intervention during their time at school during the aforementioned periods. In addition, all students, regardless of the group they are assigned to, will be evaluated using various instruments both before and after the intervention. This will be a direct evaluation by an evaluator member of the research team and through a report by their preschool educator. The parents and teachers will also be asked to evaluate certain behaviours and emotions of their children both before and after the intervention.

#### Inclusion criteria

Educational institutions that meet the following inclusion criteria will be invited to participate:

1. Municipal or subsidised educational institutions
2. Mixed educational institutions
3. Educational institutions with preschool education and one class per level
4. Educational institutions with a high vulnerability index as assessed by a School Vulnerability Index-National System of Equality Allocation (IVE-SINAE) (*Índice de Vulnerabilidad Escolar-Sistema Nacional de Asignacion con Equidad*) ≥ 75%

#### Exclusion criteria

A criterion for exclusion will be educational institutions that are already developing or implementing a manualised programme to promote social-emotional skills or participating in a similar study. This criterion is considered important if the educational institutions already have a prevention programme of this nature or are participating in a similar study, and it is possible that they have already invested time and resources in its implementation, so it would be counterproductive to ask them to incorporate another intervention programme or replace theirs. However, educational institutions may be implementing activities that promote social-emotional skills outside of a manualised programme.

### Sample size

Since this is a pilot study, it is not appropriate to calculate a sample size for establishing the effectiveness of the intervention [45]. However, we have calculated a suitable number of children for this study. According to some studies, we expect that the proportion of children who will have disruptive symptoms within 1 year in the waiting-list control group will be 30%, compared to 10% for those children in any of the intervention arms. For a loss to follow-up calculated at 20% and in a two-sided contrast analysis, 75 children will be required per arm and 225 children in total. Despite evidence to support a one-sided analysis for this case, since we assume that the intervention will not cause harm, we will conduct a two-sided analysis as is common in randomised controlled trials. This number is adequate according to recommendations for feasibility studies that propose a minimum of 30 participants per arm to estimate the parameters for future sample size calculations [46]. We have considered the selection of 12 educational institutions as an adequate size for this pilot study, with three arms: four schools in the control group and four in each of the two intervention groups. In one of the intervention groups, the ICPS programme facilitator will be the preschool educator of the educational establishment itself, while in the other group, the facilitator will be an external preschool educator hired by the research team. Since each of these educational institutions meet the aforementioned inclusion criteria, they may be considered to have similar characteristics. An average participation of 20 students is desired per course, so we hope to recruit a total of 20 students per school. Each arm of the study should have a total of 80 students for a total of 240 students, just above the expected sample size calculated.

### Procedures

#### Recruitment

**Fig. 2:**
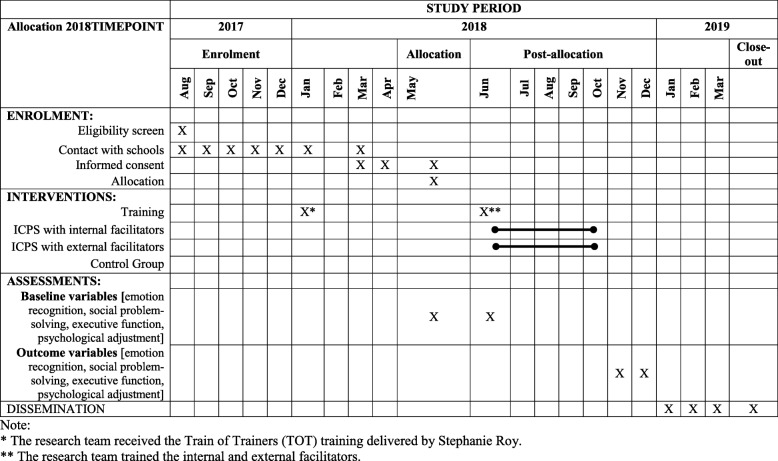
Flowchart

The Fig. 1 and the flowchart displayed in Fig. 2 summarises the recruitment procedure. A list of educational institutions of four municipalities of the province of Santiago will be requested from the Ministry of Education for Estación Central, Peñalolén, Quilicura, and Lo Espejo. These four areas will be selected according to the already-established good relationships between the educational authorities and the San Carlos de Maipo Foundation. A selection of educational institutions will be made according to the inclusion criteria. Then, a member of the research team will contact the authorities of the selected schools by telephone or email and the inclusion and exclusion criteria will be confirmed. A pre-selection of educational institutions will be done, and information will be emailed to them along with an invitation. If necessary, a meeting will be held with the authorities of the educational establishment to explain the study details. The first 12 schools that agree to participate according to the conditions will be selected for the study. If less than 70% of the expected number of participating schools (fewer than nine schools) agree, another municipality with all eligible schools will be included and contacted until we reach 12 schools. If 10 or more schools from the four municipalities agree to participate, no more municipalities will be included in the sample frame. These procedures will be followed for practical, timing, and economic reasons.

After obtaining the signed authorisation of the principal of the educational establishment, the parents will be informed of their participation in the study. A sealed envelope will be sent to the parents containing information on the study, the informed consent form, and the questionnaires to answer. The signed parent responses must be returned to the head teacher.

#### Randomisation

Once the educational institutions have been recruited and the informed consent forms have been sent to the parents, each school will be randomly placed in the intervention groups or the control group. This process will be done by a collaborator of the team. Four educational institutions will be randomly placed in each intervention group and four will be placed in a control group. The educational institutions will then be informed of their assigned group. Subsequently, the baseline or pre-intervention evaluation will be performed.

#### Blind condition

Since the main measurements include questions that are specific to the intervention, it is not possible for these to be blind measurements. However, for the secondary measurements, the team of evaluators will be blind to the groups that the schools and students were assigned to.

### Recruitment of facilitators

The professionals in charge of the intervention will be preschool educators who have at least 2 years’ experience in classroom work with children in preschool or kindergarten. They will participate in a 16-h training programme. We expect to train 10 facilitators. Eight will be teachers who are already working with preschoolers in the selected schools and two will be hired as members of the research team.

### Intervention group: ICPS

A manualised intervention will be conducted using I Can Problem Solve by Dr. Myrna Shure. ICPS content includes vocabulary and concepts about emotions and emotion recognition, the development of social problem-solving skills, practising alternative solutions, consequences, and sequential thought (solutions-consequences) using interactive techniques (for example, games, role-playing, and the use of stories, illustrations, blocks, animal figurines, and puppets), and guided discussion strategies used to solve problems. These activities are organised in 59 sessions of 20 min each delivered three times a week by trained early teachers over 5–6 months. The ICPS programme will be adapted from the English version, through the translation, editing, and adaptation of all of the components of the ICPS preschool programme. These activities will be carried out during the first 6 months of the study. Team members have an advanced command of English and have experience in the cultural adaptation of other programmes with similar characteristics. The following procedure was followed:

1. The manual of the ICPS programme was translated from English to Spanish by a certified translator
2. This translation was adapted linguistically and in its content by two members of the research team using a blind review by each
3. Once a first draft of the manual was generated, it was reviewed by one experienced preschool educator working with the research team. This aimed to incorporate the perspective of the future users of the manual in the adaptation process
4. Many cultural aspects have been considered in the adaptation of the programme:
  - Adaptation of activities to class size. The ICPS sessions or games were originally proposed to be implemented in groups of 10 to 15 students, but the class size in Chile is around of 30 students. Therefore, all the activities were planned to include the participation of the whole class. For example, activities offer different roles to the students during the games to assure that every student has something to do
  - Class management. Additional material of class management was included in the manual, providing positive behavioural strategies to facilitate the work with the whole class and promoting motivation and attention
  - Active participation of all adults in the classroom. Preschool classes in Chile have normally two adults working with the children: the certified early teacher and a teacher assistant. The new adapted manual incorporated instructions to both adults in order to engage the full participation of these adults in the intervention and help with the class management during the games
  - Drawings and material were culturally adapted. All pictures of the manual were revised and changed to be appealing to children living in Chile in order to facilitate to emotional connections from the students to the characters
  - Format and material. Most of the original sessions used white-and-black drawings on paper to perform the activities with children. In order to engage children and increase motivation, we have introduced colour to the drawings, actual pictures of children (e.g. children expressing different emotions), and all these images were included in keynotes presentations for the children to present to the whole class, in addition to the images on paper. Additionally, we have increased the number of activities where children could interact with the facilitator and with other children, especially using puppets
5. The entire process was supervised directly by the research team of Dr. Myrna Shure, author of the programme. Regular meetings were held (every 2 weeks) throughout the initial adaptation process and during the pilot programme

The intervention is designed for two different groups, students and preschool educators/assistants.

#### Students

Students will participate in the ICPS programme that has been adapted to the Chilean culture. This programme is a universal intervention designed to promote interpersonal cognitive processes and problem-solving skills in children from preschool through sixth grade. This project will adapt the preschool programme, which has a total of 59 sessions. Each session lasts approximately 20 min. The trained facilitator follows a simple manual that guides their work with the students on ICPS vocabulary and concepts and the development of problem-solving skills such as practising alternative solutions, consequences, and the sequential thought (solutions-consequences). Interactive techniques and guided discussion strategies are used to solve problems. The interactive techniques include games, role-playing, and the use of stories, illustrations, and puppets. These may also be used in other curricular activities for children, whether they are working on math, reading, or science. Children learn *how* to think, not *what* to think.

#### Preschool educators/assistants

The educators/assistants from the selected schools and the facilitators who are part of the research team for the intervention will be trained in the programme during a 2-day session (8 h per day). This will be provided by coaches previously trained by Stephanie Roy, official coach of the ICPS programme. In addition, supervision will be conducted with a member of the research team twice a month in order to solve issues involving the programme’s content and implementation.

### Control group

The educational institutions assigned to the control group will continue to carry out their normal academic and prevention activities. Although one initial exclusion criterion was that the educational institutions not be implementing a manualised programme for the development of social-emotional skills at the time they agree to participate in this study, these schools will be able to continue with their normal activities. The normal academic and prevention activities follow the curriculum designed by the Chilean Ministry of Education, which is common to all state and subsidised schools in Chile. This curriculum for pre-kindergarten includes basic literacy and language learning (e.g. letter/sound recognition and production, number and shapes recognition, and production) and prevention activities, such as healthy nutrition and hygiene habits, and social and emotional teaching (emotion recognition, and relationship skills). However, the social and emotional teaching is less structured, and it does not follow a clear and manualised programme such as ICPS.

### Follow-up

All of the participating students, teachers, and parents will be evaluated during the same period of time after inclusion in the study. For the schools in the three arms of the study, the following times will apply for the quantitative evaluations: pre-intervention (beginning of the school year) and post-intervention (end of the school year) for all of the classes participating in the study.

Qualitative evaluations of the intervention will be performed through interviews, surveys, or group meetings (focus groups) in the middle and at the end of the programme’s implementation. During these same activities, the different measurements used in the pre-intervention and post-intervention periods will be used for evaluation.

### Outcomes measures, instruments, and other measures

#### Demographic features

A brief instrument will be designed to collect data from children and parents regarding demographic variables (such as age and gender).

#### Primary outcomes

##### Acceptability

The acceptability will be evaluated by establishing how this intervention programme is received by students, parents, teachers, and authorities of educational institutions and to what extent this intervention responds to the needs of this target population.

Specifically, acceptability will be evaluated using a questionnaire that will be answered after each session by the facilitator. It will include questions regarding the fidelity of the implementation (e.g. ‘How many sessions were delivered during the week?’ ‘Did you implement all of the activities for each session?’ ‘How many minutes did you use to implement the session?’), and the participation of the children and the class climate during the sessions (e.g. the motivation, attention, and degree of involvement in all of the activities, and understanding). Participation and class climate will be rated using a scale from 1 = low to 5 = high. All of the sessions will be filmed in their entirety to assess the fidelity of each intervention and children participation by an independent observer, using similar to the above questions. Having the same questions answered by facilitators and video observers will help to recognise the degree of a facilitator’s self-awareness of her performance, important skill to facilitate change and improvement over time and with the help of supervision. More than 50% of the recorded sessions, randomly selected, will be observed and analysed by trained evaluators using a pre-designed form where they will register and answered the following questions: (1) Were the activities performed as they were stated in the manual?; (2) Did the facilitators use and follow the scripts of the activities?; (3) Did the facilitators motivate students for participation according to the manual?; (4) Did the facilitators use the ICPS vocabulary during the sessions?; and (5) Did the facilitators pronounce the ICPS words with the emphasis and stress suggested by the manual? All the above questions will be rated using a scale from 1 = never to 5 = always. The recorded sessions will also be used to assess the process of building the relationship between the facilitator and the students: (1) Did the facilitator treat and show respect to the students; (2) Did the facilitator give space and time for the participation of all students (including those with special educational needs and those introvert students); and (3) Did the facilitators consider and view children as an active agents of their own learning? All the above questions will also be rated using a scale from 1 = never to 5 = always.

The facilitators will have a supervision meeting with a member of the research team twice a month where they will answer a brief questionnaire regarding the acceptability of the intervention (e.g. ‘Was this session’s activity interesting/relevant?’ ‘Did you like the session?’ ‘What did you like the most?’ ‘What did you like the least?’), and opinions about potential changes to be included for the future (e.g. ‘In your opinion, is there something you would change/replace/include?’). The answers to these questionnaires will be used continuously to improve the sessions if needed.

There will also be a brief assisted survey for the students, a self-reported survey for the teachers who are not implementing the programme, and a self-reported survey for the parents asking about the acceptability of the programme.

##### Feasibility

The feasibility and viability will be evaluated by measuring the achievement of the study’s objectives and by making a detailed assessment of whether it is possible to develop an effective study on a larger scale. The recruitment and the intervention process will also be evaluated. For this purpose, data will be collected on the number of schools that are eligible, those that are contacted, and those that agree to participate. Data will also be collected on the number of students, parents, and teachers contacted and those who consent and agree to participate. Data will also be collected on the number of sessions attended (by the students, teachers, and parents), the time needed to complete the questionnaires and student assessment tests, and the loss of participants during follow-up.

Data will also be collected on the recruitment process for the facilitators and their participation in the training.

#### Secondary outcomes

1. The Minnesota Executive Function Scale (MEFS) [47, 48]. This instrument is used to evaluate the executive functions; specifically, cognitive flexibility, working memory, and inhibitory control, among individuals beginning at age 24 months and extending throughout the lifespan. It is an adaptive virtual card-sorting task delivered on a tablet (2–6 min; 4-min average test duration). The MEFS has been used with more than 17,000 individuals and has been found to be reliable [47] and valid [49]. It is normed on a representative sample of 7410 typically developing children aged 2–13 years, and 553 adults. This measure also has been validated in at-risk preschoolers [50, 51]. It is related to emotional understanding in preschoolers [35]. The MEFS is sensitive to training interventions [52], especially in low-income children [53]
2. The Assessment of Children’s Emotions Scale (ACES) [54]. The ACES consists of three sub-scales: facial expressions, social situations, and social behaviour. In this study, only the facial expressions sub-scale will be used to evaluate emotion expression knowledge and whether the subjects exhibit any anger bias. The 26-item sub-scale consists of colour photographs of ethnically diverse elementary schoolchildren depicting four expressions of each of the four basic emotions (happy, sad, angry, and scared) and 10 images of children without obvious facial expressions [55, 56]. The examiner shows the child the photographs one at a time and each time asks: ‘Is the child in the picture happy, sad, angry, or scared?’ Then the examiner registers the child’s answer. The emotion accuracy score reflects how many items the children answer correctly, and the anger bias score is the percentage of time the children incorrectly identify the faces as displaying anger [37]
3. The Challenging Situations Task (CST) [57]. This instrument evaluates the ability of children to solve social problems. The children are presented with six vignettes that describe problems between peers. Following the presentation of each challenging situation, four pictures of happy, sad, angry, and neutral affect are presented in random order and labelled for the child. Then, the child is asked to point to the picture that best describes the answer to ‘How do you feel when (this situation) happens to you?’ [57]. Then, four pictures of behavioural responses (prosocial, aggressive, manipulation of others’ feelings, and avoidant) are presented in random order and the child is asked ‘What do you do (in this situation)?’ The answers are categorised into four possibilities: (1) prosocial, (2) aggressive, (3) crying, and (4) avoidant. Scores for affective and behavioural responses used are the number of times each affect and each behavioural response is chosen by each child across the six situations [57]
4. The Strengths and Difficulties Questionnaire (SDQ) [58] is widely used in Chile [59, 60]. This 25-question questionnaire explores different symptoms grouped into five sub-scales (with five items each): (1) emotional symptoms, (2) behavioural problems, (3) problems with peers, (4) symptoms of lack of attention and hyperactivity, and (5) prosocial skills. The first four sub-scales refer to difficulties that children may have and may be grouped together in a general sub-scale of difficulties (20 items). The sub-scale of prosocial skills refers to positive and adaptive behaviours in relationships with others. Each item is answered on a scale of responses from 1 = not true to 3 = absolutely true. There is a version for teachers, parents (to evaluate children from 4 to 16 years old), and a self-report for teenagers (ages 11 to 16 years old). It has been widely used [60–64] and has shown good psychometric characteristics [65]. The teachers’ and parents’ version of this instrument will be used

In all cases, we will register the time and resources consumed for children and parents’ assessments in order to gain information for planning the future, larger randomised controlled trial.

#### Data management

After the examiners have completed the assessments of children, and parents and teachers have sent back the questionnaires, the data will be entered by trained research assistants into a secure platform, without identifying information (each participant will be assigned an ID number). The original copies of the answer sheets and questionnaires will be filed and stored, under lock and key, in the Principal Investigator’s (PI’s) office, along with the list linking the participants’ names and ID numbers. Only two research assistants, in charge of data entry, and the statistician will have access to the database. All the recordings will be stored, under lock and key, in the PI’s office.

### Data analysis

All the data will be collected by trained research assistants and stored and organised by the project coordinator. All of the forms will be typed and then securely stored in a warehouse. All of the data will be handled confidentially and only the PI at the University de los Andes and the PI of the San Carlos de Maipo Foundation will have access.

#### Qualitative data analysis

For the interviews, two researchers will independently code the information and identify the relevant topics. Any difference in the analyses by these two researchers will be discussed and resolved. If it is not possible to reach an agreement, a third researcher will participate in the discussions to reach a solution.

For the analysis of the video recordings, two adherence evaluators will independently code the data and the prevailing topics in these will be identified from the evaluated videos. Any difference in the analyses by these two researchers will be discussed and resolved. If it is not possible to reach an agreement, a third researcher will participate in the discussions to reach a solution.

The results of the satisfaction surveys will be used to improve the content and implementation of the interventions.

#### Quantitative data analysis

The presentation of the results of this pilot study will be guided by the Consolidated Standards of Reporting Trials (CONSORT) for randomised controlled trials. Intention to treat will be used as the basis for the analysis of all of the participants in both groups. Initially, the sample will be described and the variables will be presented for the different follow-up events. To evaluate the changes produced by the intervention, analyses will be performed with regression models—simple for the continuous dependent variables and logistic for the dichotomous dependent variables. ‘Group’ will be used as the main independent variable. It will be controlled by the pre-intervention evaluations and the analyses will be adjusted by clustering given the hierarchical nature of the participants (students in a school). Exploratory analyses will be performed to evaluate the effect of different variables on the intervention results. Finally, sensitivity analyses will be performed to explore the effect of the data lost in the results. All analyses will be performed using complete data.

#### Trial management

The study will comply with local Research Governance requirements.

## Discussion

This study is the first to assess the acceptability and feasibility of the I Can Problem Solve programme in a Spanish-speaking country in Latin America. The result of this pilot study should provide valuable information to plan and conduct a larger study in the future to test the effectiveness of the programme to reduce behavioural problems and psychological distress among preschoolers.

Promoting social and emotional learning skills may produce immediate benefits for the children and their families, reducing behavioural problems, but we also expect long-term effects improving academic performance, mental health, the school climate, and a reduction of risk behaviours.

### Limitations

There are some potential risks. It may be difficult to recruit schools to conduct the pilot, especially if we consider that we are introducing a new programme to be implemented along with the usual curriculum, and we may face some resistance. Additionally, the study design may be less appealing to schools because they may prefer implementing already scientifically effective interventions. However, given the fact that there are no available interventions similar to the one proposed herein, participation is anticipated. To minimise this risk, we will prepare the recruitment carefully and inform the schools in a timely manner utilising the substantial networks of the members of the Chilean team.

Another potential difficulty may be keeping the facilitators motivated to deliver the intervention over the entire academic year. We have planned regular meetings where it will be possible to assess the adherence to the intervention and the workload.

## Trial status

The recruitment began in October 2017 with the permission of the local educational authorities.

## Additional file

Additional file 1: Standard Protocol Items: Recommendations for Interventional Trials (SPIRIT) 2013 Checklist: recommended items to address in a clinical trial protocol and related documents. (DOCX 61 kb)

## Abbreviations

ACES: Assessment of Children’s Emotional Skills
CASEL: Collaborative for Academic, Social, and Emotional Learning
CC: Control nursery-control kindergarten
CONSORT: Consolidated Standards of Reporting Trials
CST: Challenging Situations Task
CT: Control nursery-trained kindergarten
HPSB: Hahnemann Preschool Behaviour Scale
ICPS: I Can Problem Solve
IVE-SINAE: School Vulnerability Index-National System of Equality Allocation (*Índice de Vulnerabilidad Escolar-Sistema Nacional de Asignacion con Equidad*)
MEFS: Minnesota Executive Function Scale
PATHS: Promoting Alternative Thinking Strategies
PI: Principal investigator
PIPS: Preschool Interpersonal Problem-Solving
SDQ: Strengths and Difficulties Questionnaire
SEL: Social and Emotional Learning
TC: Trained nursery-control kindergarten
TT: Trained nursery-trained kindergarten
WHNG: What Happens Next Game
